# Aerogel-Lined Capillaries as Liquid-Core Waveguides for Raman Signal Gain of Aqueous Samples: Advanced Manufacturing and Performance Characterization

**DOI:** 10.3390/s24185979

**Published:** 2024-09-14

**Authors:** Felix Spiske, Lara Sophie Jakob, Maximilian Lippold, Parvaneh Rahimi, Yvonne Joseph, Andreas Siegfried Braeuer

**Affiliations:** 1 Institute of Thermal, Environmental and Resources’ Process Engineering (ITUN), Technische Universität Bergakademie Freiberg (TUBAF), 09599 Freiberg, Germany; 2 Institute of Nanoscale and Biobased Materials (INBM), Technische Universität Bergakademie Freiberg (TUBAF), 09599 Freiberg, Germanyyvonne.joseph@esm.tu-freiberg.de (Y.J.)

**Keywords:** liquid-core waveguide (LCW), Raman spectroscopy, aerogel, liquid-core optical fiber (LCOF), aerogel-lined capillary

## Abstract

An advanced process for the manufacturing of aerogel-lined capillaries is presented; these are applicable as liquid-core waveguides for gaining the Raman signal of aqueous samples. With respect to the spin-coating process we have used so far for the manufacturing of aerogel-lined capillaries, the here-presented manufacturing process is advanced as it enables (i) the lining of longer capillaries, (ii) the adjustment of the lining-thickness via the lining velocity, and (iii) the reproducible generation of crack-free linings. The key parameters of the advanced process and their effect on the fabrication of aerogel-lined capillaries with optimal Raman signal gain are reported and related to the thickness and topography of the aerogel linings by the support of scanning electron microscopy.

## 1. Introduction

Linear Raman spectroscopy is a molecule-specific, quantitative, noninvasive, and fast spectroscopic technique suitable for real-time and online spectroscopic analysis of aqueous samples. Therefore, linear Raman spectroscopy (RS) is a promising tool in process analytical technology (PAT) [1,2,3,4,5,6,7,8,9,10]. Unfortunately, due to the small Raman scattering cross-section, either high excitation laser powers or long signal integration times are required to lift the signal above the noise. In many cases, neither of them can be increased arbitrarily [9,11,12]. In addition, the use of high laser power can disqualify RS for use in explosion-proof areas of industrial plants [7]. Some measures can be taken to obtain sufficiently high Raman signals at low excitation laser power [13,14], which do not always qualify for industrial environments. In the case of liquid samples, the use of liquid-core waveguides [13,15,16,17,18,19,20,21,22,23,24] (LCWs) enables just that.

**Figure 1:**
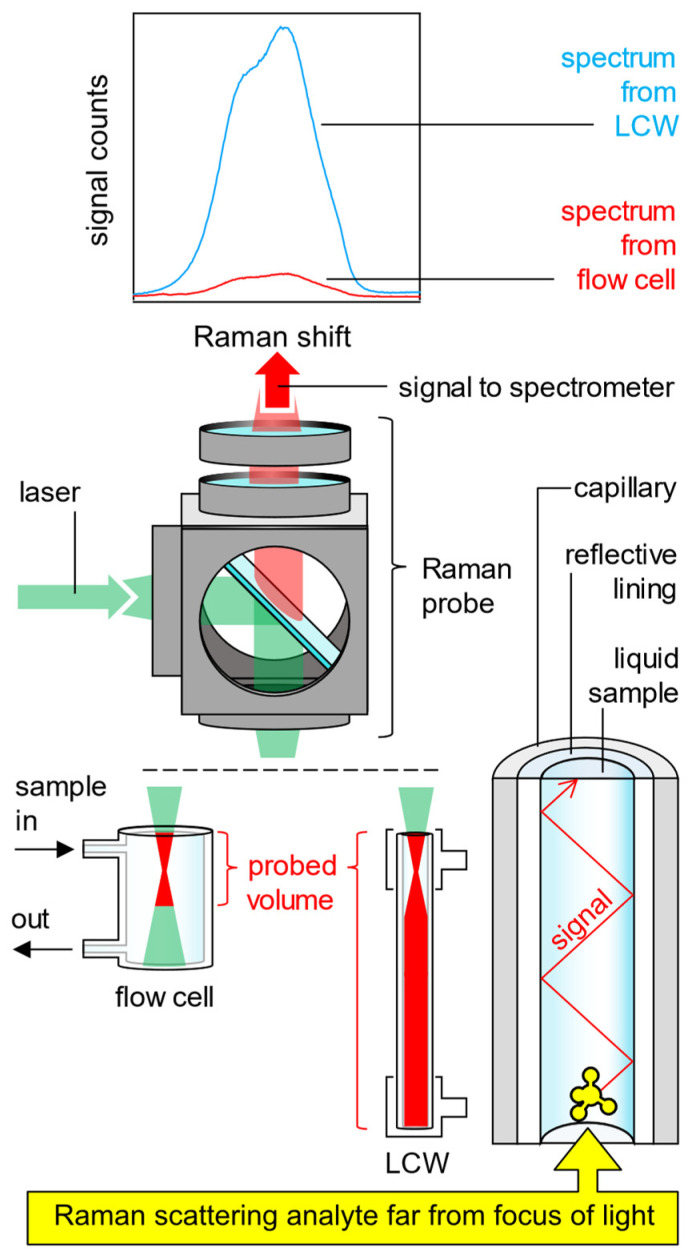
Liquid-core waveguides gain the Raman signal of liquid aqueous samples by enhancing the probed volume due to light guidance via total internal reflection (bottom right). From a Raman probe (center), the green excitation light can be focused either into a flow cell (bottom left) or into an LCW (bottom center). The water stretch Raman spectra recorded from the flow cell or from the LCW are compared (top).

As shown in Figure 1, below right, an LCW can be a capillary with a hollow core through which the laser excitation light, the Raman scattered light, and the liquid sample are guided simultaneously. The propagation of light along the liquid core is possible because the capillary is lined on its internal surface, either with a highly reflective metal (mirror) or with a material that enables the total internal reflection (TIR) of light at the interface with the liquid in the hollow core of the capillary. The main advantage of TIR is that the entire spectrum of the incident light, for which the condition of TIR is fulfilled, is reflected totally. The reflectivity of a reflective metal coating, instead, is not total and, additionally, is a function of the wavelength. The excitation-laser-photons, which are coupled into the one end of the liquid core of the LCW, are guided along the liquid core until they exit at the other end of the LCW. Also, the Raman scattered photons, which emerge inside the entire volume of the liquid core due to inelastic light/matter interactions, are guided to the ends of the LCW, from where they can be collected and analyzed in a spectrometer. In contrast, spectroscopy in a transparent flow cell almost exclusively detects Raman-scattered photons from the tiny volume of the focal point of the detection optic. Figure 1 compares the probed volume when measuring in a flow cell (bottom left in Figure 1) with the probed volume when measuring in an LCW (bottom center in Figure 1). 

This comparison shows that replacing a transparent flow cell with an LCW makes it possible to probe a larger (longer) volume with the same Raman probe at the same excitation laser power, thus detecting more Raman signal (top in Figure 1) from the liquid sample and achieving a better signal to noise ratio. LCWs are not only used in Raman spectroscopy [17,20,22,23,25,26,27], but also in other spectroscopic techniques, such as absorption spectroscopy [28,29,30,31,32,33,34,35,36] or fluorescence spectroscopy [37,38].

The highly reflective metal lining can be a silver or gold mirror [39], for example. For TIR, materials such as Teflon AF [18,21,22,23,40,41,42], which feature a lower refractive index (n) than the aqueous liquid core, can be used (1.29 = n_TeflonAF_ < n_water_ = 1.33). Unfortunately, depending on the chemical properties of the liquid sample in the hollow core of the LCW, the reflectivity of the respective lining can be impaired by the deposition of microorganisms (fouling), salts (scaling), or corrosion on the lining. If the selected lining is fluorescent [23], complex background subtraction in the Raman spectra may also be required. One solution to these numerous challenges is to surround the flowing liquid sample with ambient air instead of a reflective material or a solid material with a low refractive index (optofluidic jet waveguide [15,16]). Then the light is guided through the sample by TIR (as when using Teflon AF) between the liquid jet and the surrounding air. Unfortunately, optofluidic jet waveguides cannot be used in industrial applications, where online monitoring in “closed” systems is preferred. However, the principle of an optofluidic jet waveguide can be imitated by surrounding the flowing liquid sample with hydrophobic silica aerogel instead of ambient air, i.e., with a solid material that can consist of more than 99 percent air by volume [43,44,45,46,47,48]. Silica-based aerogels feature a refractive index similar to that of air (n_aerogel_ = 1.1 < n_water_ = 1.3) [49]. Therefore, a liquid sample which flows through an aerogel-lined capillary is surrounded by quasi pure air. If the highly porous aerogel-lining is made hydrophobic, liquid aqueous samples, which represent the liquid core of the LCW, do neither wet nor penetrate the lining. The hydrophobicity also protects the transparent aerogel lining from fouling and scaling [50]. Hydrophobic silica aerogel linings are flexible and resistant to substrate bending or twisting [49].

In a previous publication [51], we showed the production of aerogel-lined fused silica capillaries via spin-coating and demonstrated their Raman signal gain capability of pure liquid water and of a liquid acidic aqueous mixture of organic substances. During a seven-day exposure period of the capillaries to the aqueous mixture, the Raman signal gain capability of the capillaries was neither worsened nor was any fouling or scaling observable on the lining. Moreover, Raman spectra measured from liquid organic samples inside the hollow core of an aerogel-lined capillary neither contain signals from the silica backbone of the aerogel lining nor from the methyl groups of the hydrophobic surface of the aerogel lining.

The spin-coating process used in [51] for the manufacturing of aerogel-lined capillaries unfortunately suffers from some inherent drawbacks: (i) the in situ observation of the formation of the aerogel lining inside the capillary during spin-coating is challenging and could not be put into practice by us. This ultimately makes the optimization of the lining process difficult. (ii) Capillaries with a length of longer than 20 cm could not be spin-coated within our self-engineered spin-coater. This length limitation is disadvantageous, due to the almost linear signal gain increase with capillary length in the range of 2 to 20 cm.

In consequence, we, here, present an advanced technique for the production of aerogel-lined capillaries, which does not suffer from the abovementioned drawbacks. It enables the lining of longer capillaries (here demonstrated up to 100 cm) and enables the visual observation of firstly lining the capillary with the alcogel dispersion and secondly drying the alcogel lining to the aerogel lining. The linings are characterized considering their Raman signal gain performance and their topography using scanning electron microscopy. We finally describe how the parameters of the manufacturing process have to be coordinated in order to create aerogel linings featuring optimal Raman signal gain.

## 2. Methods and Materials for Manufacturing and Characterization of Silica Aerogel-Lined Capillaries

The advanced manufacturing process and the characterization of the fused silica capillaries lined with hydrophobic silica aerogel are divided into the following successive individual steps.

First, a dispersion consisting of hydrophobic, highly porous silicate particles and ethanol is prepared (alcogel dispersion). In a second step, the alcogel dispersion is pumped into a fused silica capillary. Subsequently, when the alcogel dispersion is then pumped out of the capillary again, a film of the alcogel dispersion forms on the inside of the capillary wall (alcogel lining). The alcogel lining is then dried by passing air through the alcogel-lined capillary. This enables a controlled evaporation of the ethanol from the pores of the hydrophobic silica particles in the alcogel lining and converts the alcogel lining into an aerogel lining.

In order to quantify the Raman signal gain of an aerogel-lined capillary, the intensity of the Raman spectrum measured from the water core inside the aerogel-lined capillary is related to the intensity of the Raman spectrum measured from bulk water inside a cuvette in the same arrangement. This comparison is identical to the comparison of the measurements made in the flow cell and the LCW that are sketched in Figure 1.

Finally, scanning electron microscopy (SEM) is employed to examine the topography of the aerogel lining as well as to measure the thickness of the aerogel lining.

### 2.1. Preparation of the Alcogel Dispersion

**Figure 2:**
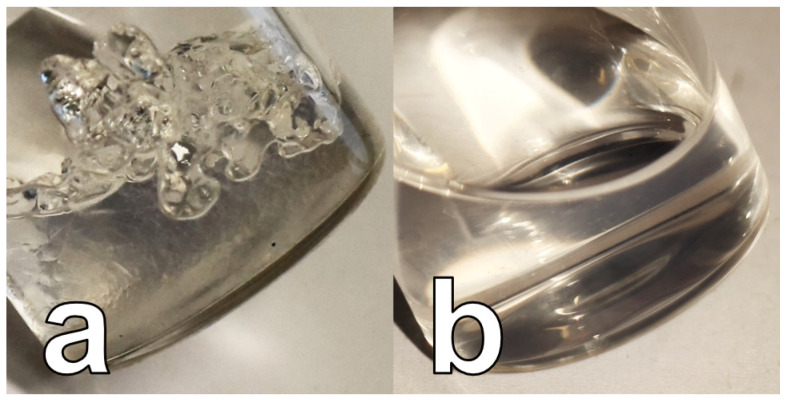
Photo of (**a**) the coarse-grained alcogel, and (**b**) alcogel dispersion after ultrasonic treatment.

The hydrophobic silica alcogel is prepared according to a recipe from Fraval [49], as we have already described in an earlier publication [51]. As specified in [51], the hydrophobization of the silica gel matrix is followed by a two-step washing procedure using ethanol. After the second step of the washing procedure with ethanol, the gel is separated from the liquid ethanol by centrifuge. The liquid supernatant is discarded. The solid residue is a coarse-grained transparent colorless to slightly blue shimmering gel (see Figure 2a) that can be further processed directly into the alcogel dispersion (as described in the following) or it can be stored in an airtight container in the refrigerator after adding fresh ethanol.

For processing the coarse-grained alcogel into an alcogel dispersion, 5.05 g of the coarse-grained alcogel, 1.00 g of ethanol, and a stirring fish are added to a 15 mL snap cap tube. The filled snap cap tube is then placed in a water bath on a magnetic stirrer. Then, the sonotrode of an ultrasonic homogenizer (Fisherbrand FB120 homogenizer, Fisher Scientific GmbH, D-58239 Schwerte, Germany) is inserted into the snap cap tube filled with the mixture of coarse-grained alcogel and ethanol. Finally, the mixture of coarse-grained alcogel and ethanol is homogenized while stirring at room temperature. The ultrasonic homogenizer operates at 80% amplitude, 3 s impulse, and 3 s pause between each pulse. The sum of pulse and pause time is 85 min. Finally, a transparent, bluish, shimmering, clear, slightly viscous dispersion is obtained (alcogel dispersion), as can be seen in Figure 2b.

### 2.2. Lining a Capillary with Alcogel Dispersion

**Figure 3:**
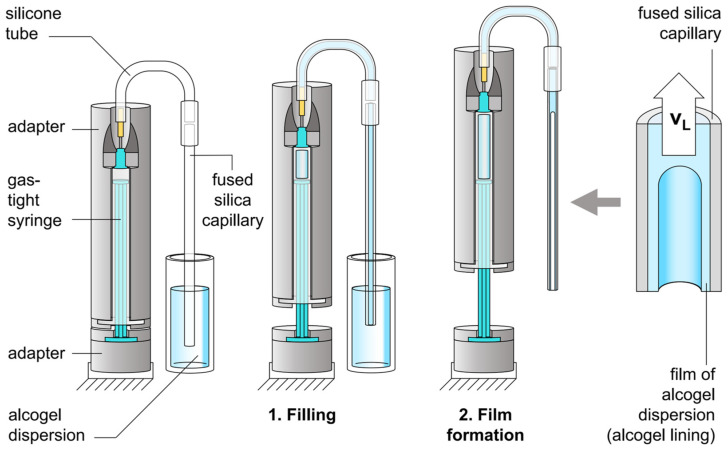
Sketch of the setup for lining a fused silica capillary with hydrophobic silica alcogel (the tensile testing machine with its sample holder and pistons is not depicted).

The lining of a fused silica capillary with alcogel dispersion is carried out as shown in Figure 3. A silicone tube is slipped over the top opening of an upright fused silica capillary (obtained from MOLEX, Lisle, IL, USA) with circular cross-section and inner/outer diameters of 700 ± 10 µm/850 ± 20 µm, respectively. The second end of the silicone tube is connected to the needle of a gas-tight syringe with a volume of 1.0 mL and an inner diameter of 4.38 mm. The gas-tight syringe is clamped, with the needle pointing upwards, into a self-engineered syringe pump, based on a modified tensile testing machine (INSPEKT mini, Meß- und Prüftechnik GmbH, 01683 Nossen, Germany). The plunger and barrel of the syringe are each inserted into self-engineered adapters, which are connected to the two pistons of the sample holder of the tensile testing machine (the tensile testing machine with its sample holder and pistons is not depicted in Figure 3). Thus, it is possible to pull apart the plunger and barrel at a defined and adjustable velocity v_M_. The lower end of the upright fused silica capillary is immersed in the alcogel dispersion. Subsequently, in order to fill the entire fused silica capillary with the alcogel dispersion, the plunger and barrel of the gas-tight syringe are pulled apart at v_M_ = 100 mm/min. As soon as the entire fused silica capillary is filled with the alcogel dispersion, the flow is stopped and the capillary is pulled out of the alcogel dispersion so that it hangs freely in the air. By then pulling the plunger and barrel further apart, the alcogel dispersion contained in the fused silica capillary is conveyed from the capillary into the gas-tight syringe. As the alcogel dispersion wets the inner wall of the capillary, a film of the alcogel dispersion remains on the inner surface of the capillary wall. An alcogel-lined capillary has been manufactured.

The mean velocity at which the alcogel dispersion flows through the capillary during its removal is called the lining velocity v_L_, specified in mm/s. The later reported lining velocities v_L_ are calculated on the basis of the inner diameter of the capillary, the inner diameter of the gas-tight syringe, and the set velocity v_M_ at which the plunger and barrel have been pulled apart.

### 2.3. Convert the Alcogel Lining into an Aerogel Lining

**Figure 4:**
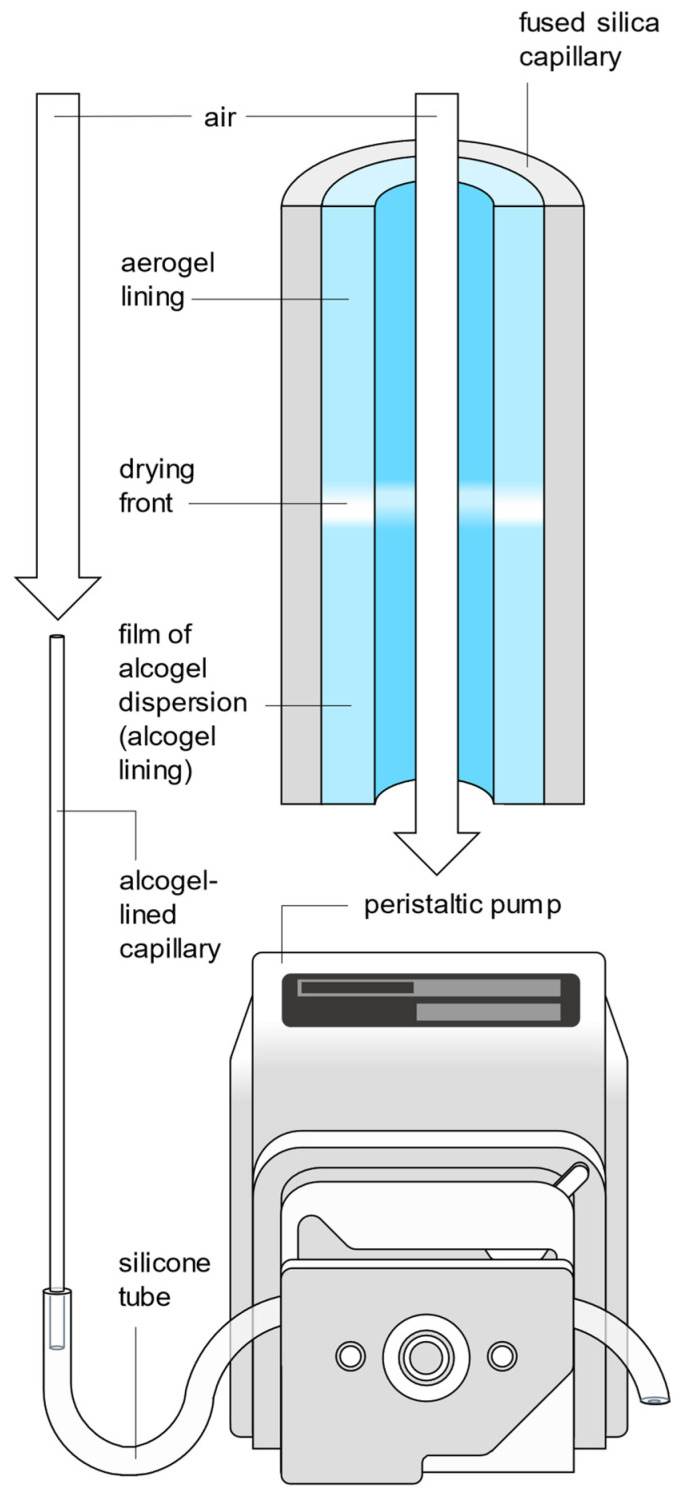
Sketch of the conversion of the alcogel lining into an aerogel lining by air drying, which is monitored via tracking the drying front passing through the entire alcogel-lined capillary during a period t_dry_.

In order to produce the desired hydrophobic silica aerogel lining of the capillaries, the liquid ethanol is dried from the alcogel lining by convective air drying at ambient pressure and temperature. For this purpose, a constant flow of ambient air is passed through the alcogel-lined capillary using a peristaltic pump (see Figure 4). The lower end of the upright capillary lined with alcogel is inserted into the silicone tube of the peristaltic pump (Dülabo PLP 380, behr Labor-Technik GmbH, 40599 Düsseldorf, Germany).

The transparent alcogel lining becomes opaque when the ethanol is evaporating and only becomes transparent again after drying, i.e., after the conversion of the alcogel lining into an aerogel lining. Therefore, an approximately 0.1 mm wide opaque drying front can be observed moving from top to bottom through the capillary (see Figure 4 and Movie S1 in the Supplementary Material) while air is conveyed through the alcogel-lined capillary. The time required for the drying front to move through the entire capillary is defined as the drying time t_dry_.

As soon as the drying front has passed through the entire capillary, the now aerogel-lined capillary is stored in the oven at 30 °C for 24 h. The aerogel-lined capillary is then removed from the oven, the oven is preheated to 220 °C, and the aerogel-lined capillary is placed again in the oven for 1 h.

### 2.4. Quantification of the Raman Signal Gain G

**Figure 5:**
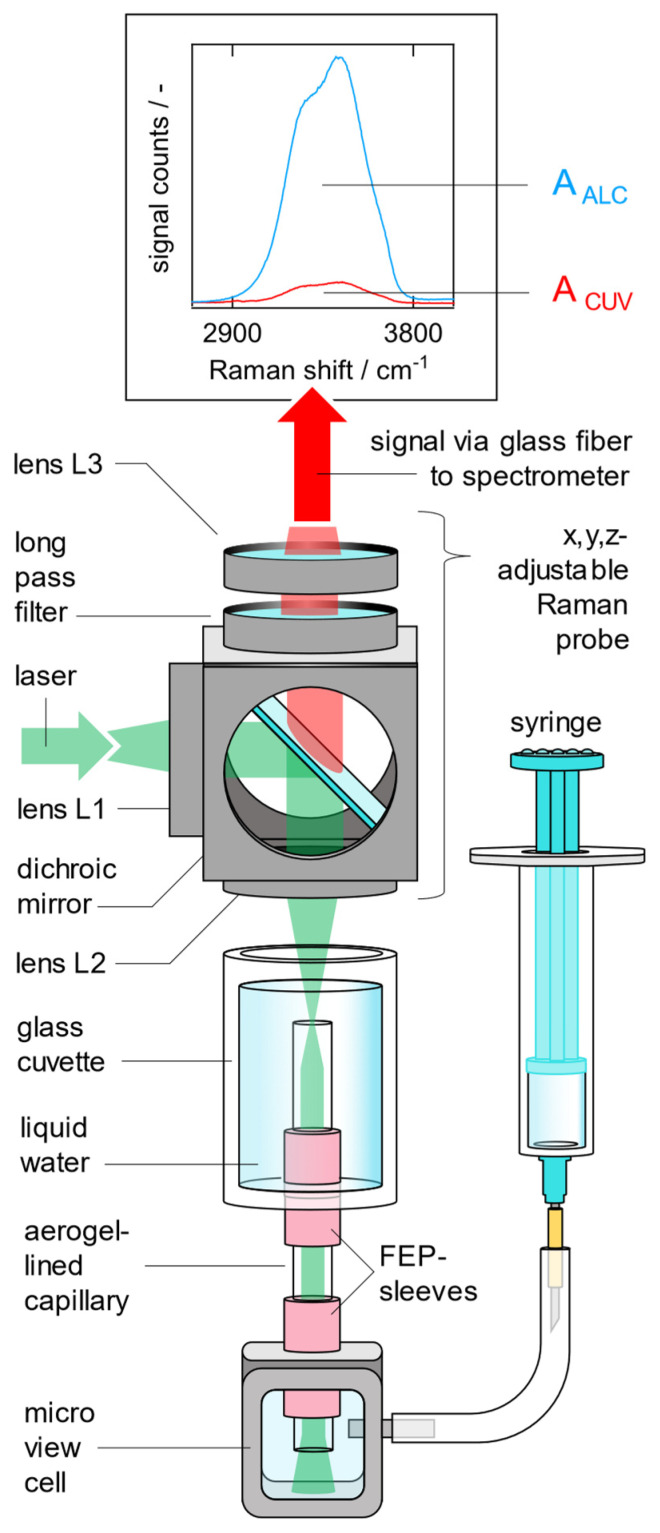
Experimental setup for measuring Raman spectra of liquid samples in the hollow core of an aerogel-lined capillary with intensity A_ALC_. In order to measure a reference spectrum with the intensity A_CUV_, the Raman probe is adjusted so that the focal point is located outside the aerogel-lined capillary but inside the water-filled glass cuvette.

In order to quantify the Raman signal gain of an aerogel-lined capillary, the setup shown in Figure 5 is used. The laser light (λ = 532 nm, P = 23 mW, Cobolt Samba 1000, HÜBNER Photonics GmbH, Kassel, Germany) is coupled into the Raman probe via a silica fiber with a core diameter of ~200 µm. Lens L1 (f_1_ = 200 mm, ∅_1_ = 50 mm) collimates the laser radiation, which is then reflected by the dichroic mirror and focused by lens L2 (f_2_ = 100 mm, ∅_2_ = 50 mm) into the cuvette which is filled with pure liquid water. As lenses L1 and L2 image the exit plane of the laser fiber with a magnification of M_L1,L2_ = −0.5 into the cuvette, the diameter of the focused laser radiation is ~100 µm. However, the aerogel-lined capillary is inserted through a drilled hole in the bottom of the cuvette, sealed by an FEP sleeve (NanoTight Sleeve IDEX H&S F-247, IDEX Corporation, Northbrook, IL 60062, USA). The lower end of the aerogel-lined capillary, also sealed by an FEP sleeve, is inserted into a self-engineered micro view cell.

The micro view cell makes it possible to visually check whether there is any air bubble at the lower end of the aerogel-lined capillary that could influence the measurement or not. The micro view cell is connected to a syringe via a silicone tube.

In order to begin the measurement procedure, a reference Raman spectrum of pure liquid water is first measured from the cylindrical, open-top cuvette (200 ms integration time). For this purpose, the x, y, z-adjustable Raman probe is adjusted so that the focal point of the laser light is not located in the hollow core of the aerogel-lined capillary, but next to the aerogel-lined capillary in the water-filled cuvette. The signals emerging from the probed volume are collected in a backscattering configuration through lens L2. The red-shifted Raman Stokes signals are purified from elastically scattered laser light, firstly, by the dichroic mirror, and secondly, by the long-pass filter (Raman razor edge RU 532 from Semrock, IDEX Corporation, Northbrook, IL, USA). The transmitted Raman signals are coupled into a detection fiber bundle via lens L3 (f_3_ = 100 mm, ∅_3_ = 50 mm). The fiber bundle contains seven glass fibers with core diameters of 100 µm. They are aligned in a round-to-linear configuration, with the round end facing the Raman probe and the linear end matching the entrance slit of the dispersive spectrometer (Ocean Optics QEPro, Ocean Insight, Ostfildern, Germany). The light-accepting area of the detection fiber bundle features a diameter of ~355 µm.

After measuring the reference spectrum, water is drawn out of the cuvette into the aerogel-lined capillary using the syringe. Due to the hydrophobicity of the aerogel lining, the liquid water inside the cuvette does not fill the capillary just due to gravity; the syringe is required as support. The x, y, z-adjustable Raman probe is then adjusted so that the focal point of the laser is located in the water-filled core of the aerogel-lined capillary. The fine adjustment of the Raman probe is performed so that the Raman band due to the symmetric stretching vibration of the water molecules in the liquid state of aggregation is maximized. Example Raman spectra are shown at the top in Figure 5. As soon as the fine adjustment of the Raman probe is complete, a spectrum is measured, with exactly the same settings at the Raman probe. The Raman signal gain

(1) $$G=\frac{A_{ALC}}{A_{CUV}}$$

is quantified as the ratio of the Raman signal intensities detected when the focal point of the laser is located inside the water-filled aerogel-lined capillary (A_ALC_) and when the focal point of the laser is located outside the aerogel-lined capillary but inside the water-filled glass cuvette (A_CUV_). The Raman signal intensities A_ALC_ and A_CUV_ are each defined as the integral of the corresponding Raman spectrum from 2900 to 3800 cm^−1^.

### 2.5. Characterization of the Thickness and the Topography of an Aerogel Lining

**Figure 6:**
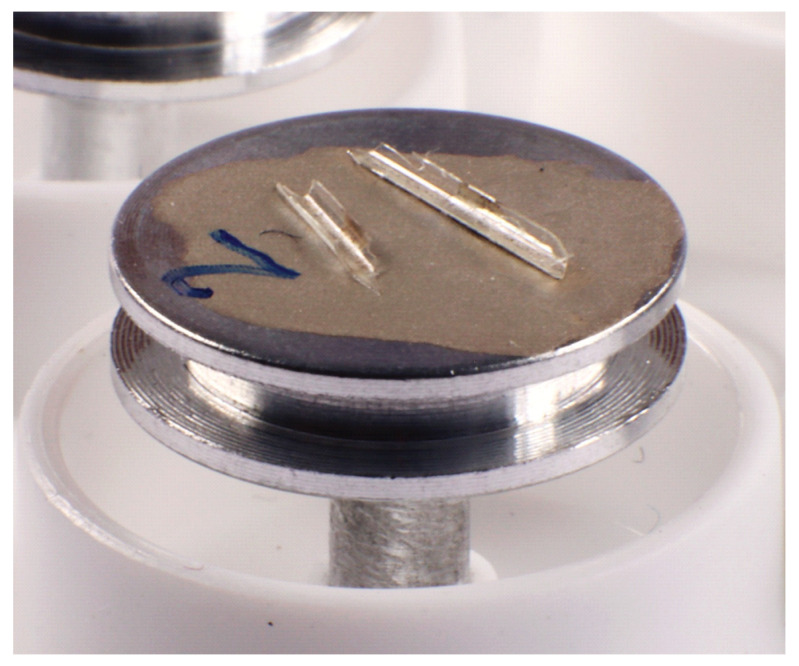
Pieces of an aerogel-lined capillary prepared for SEM.

The study of the topography and the measurement of the thickness of the aerogel linings are performed on a scanning electron microscope (PHILIPS XL30 ESEM, 22335 Hamburg, Germany). To enable this, the aerogel-lined capillaries are broken into pieces approximately 0.5 cm long, having semicircular cross-sections and, thus, presenting the surface and the breaking edges of the aerogel lining accessible for SEM. The capillary pieces are then glued to the sample holders with conductive silver lacquer (see Figure 6). After the lacquer has dried overnight, the aerogel linings of the capillary pieces can be examined under the scanning electron microscope.

**Figure 7:**
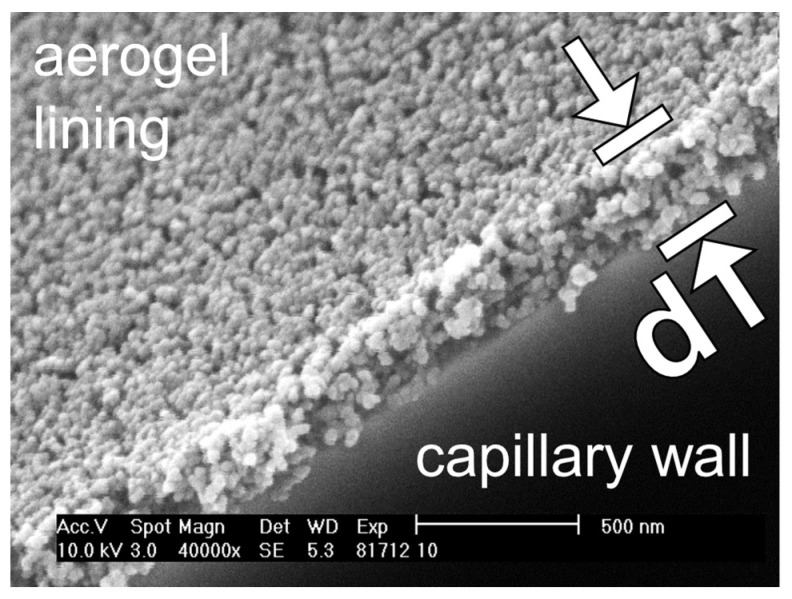
SEM image of a hydrophobic silica aerogel lining (featuring the thickness d) on the inner wall of a broken capillary.

An example SEM image is shown in Figure 7. It should be emphasized that the SEM image shows a network of particles, which looks very similar to the networks known from aerogel monoliths [52,53,54]. This is surprising, as in our case, the aerogel lining forms after drying from an alcogel dispersion, in which the alcogel particles are initially dispensed in the solvent ethanol. However, in [55], it is reported that the pore volume and surface area of porous silica films produced by a process similar to the one we present here are comparable to those of aerogel monoliths [56].

Moreover, in Figure 7, it is shown that the SEM images are suitable for measuring the thickness d of the aerogel lining. In the following diagrams, a measured lining thickness d (data point) is the mean value of about sixty individual measurements on five different fragments of one and the same aerogel-lined capillary. The error bars represent the single standard deviation of these 60 measurements of d.

## 3. Results of the Process Optimization

In the course of optimizing the manufacturing process described in Section 2.1, Section 2.2 and Section 2.3, the influence of the lining velocity v_L_ and the drying time t_dry_ on the aerogel lining thickness d and the Raman signal gain G was analyzed. For this purpose, eight individual aerogel-lined capillaries of length L = 20 cm were manufactured as described in Section 2.1, Section 2.2 and Section 2.3, and then characterized according to the methods described in Section 2.4 and Section 2.5. The eight capillaries were lined at individual mean lining velocities v_L_ of 1, 2, 3, 5, 7, 10, 13, and 20 mm/s (rounded to integer values).

**Figure 8:**
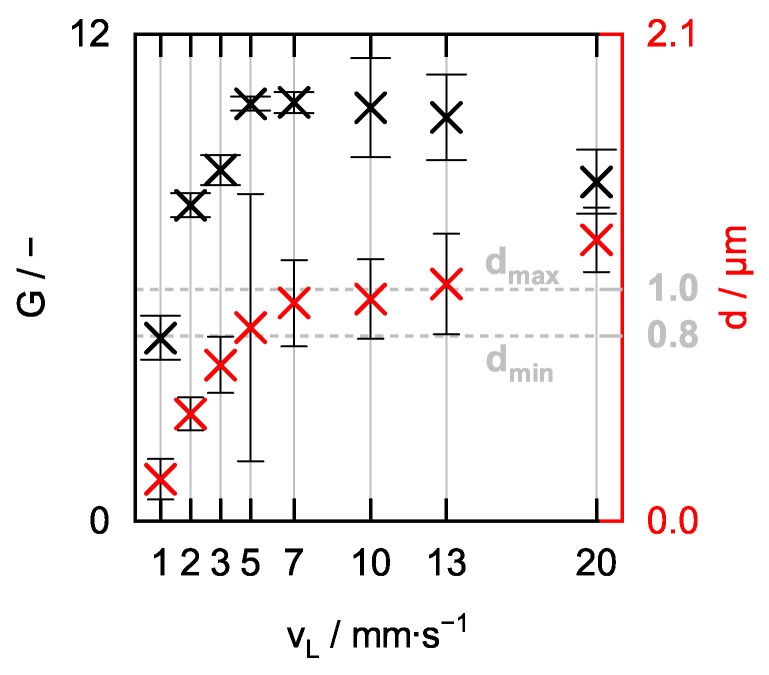
Raman signal gain G and aerogel lining thickness d as functions of the lining velocity v_L_.

In Figure 8, it is shown how the Raman signal gain G and the aerogel lining thickness d of the eight capillaries behave as a function of the lining velocity v_L_. The Raman signal gain G, presented here, is the arithmetic mean of six individual measurements, between each of which the aerogel-lined capillary was rinsed with fresh water to flush out any gas bubbles. Over the course of the six individual Raman measurements performed in the eight individual capillaries, we did not observe a tendency for G to decrease, indicating that an aerogel-lined capillary has the required durability not only for one single measurement but also for a series of measurements, as it is required for online monitoring of chemical reactions. The error bars in Figure 8 represent the single standard deviation of these six measurements.

It can be seen that an increase in the lining velocity v_L_ in the investigated range of v_L_ = 1 to 20 mm/s leads to an aerogel lining with a higher thickness d. Anticipating that the thickness of the aerogel lining after drying is proportional to the thickness of the alcogel lining before drying, this observation is in agreement with [57], which for liquids also reports an increasing liquid-film thickness with increasing lining velocity. The maximum Raman signal gain G is achieved when the lining velocity is in the range of v_L_ ≈ 5 to 10 mm/s, with the aerogel lining then having a thickness of d ≈ 0.8 to 1.0 µm. It is therefore concluded that an optimum Raman signal gain G is obtained when the aerogel lining has a thickness between d_min_ ≈ 0.8 µm and d_max_ ≈ 1.0 µm. The aerogel-lined capillary delivering the highest value of G = 10.3 ± 0.3 features a thickness of d = 0.94 ± 0.19 µm and was produced applying a lining velocity of v_L_ = 7 mm/s. However, the application of v_L_ = 7 mm/s will only produce an aerogel-lined capillary featuring an optimum lining thickness and, thus, an optimum Raman signal gain if the alcogel dispersion used for the production of the lining is prepared according to the recipe in Section 2.1. Slight changes in the mass ratio of ethanol and coarse-grained alcogel lead to a change in the viscosity of the resulting alcogel dispersion, making it necessary to adjust the lining velocity [58] in order to obtain an aerogel-lined capillary featuring the optimum lining thickness.

**Figure 9:**
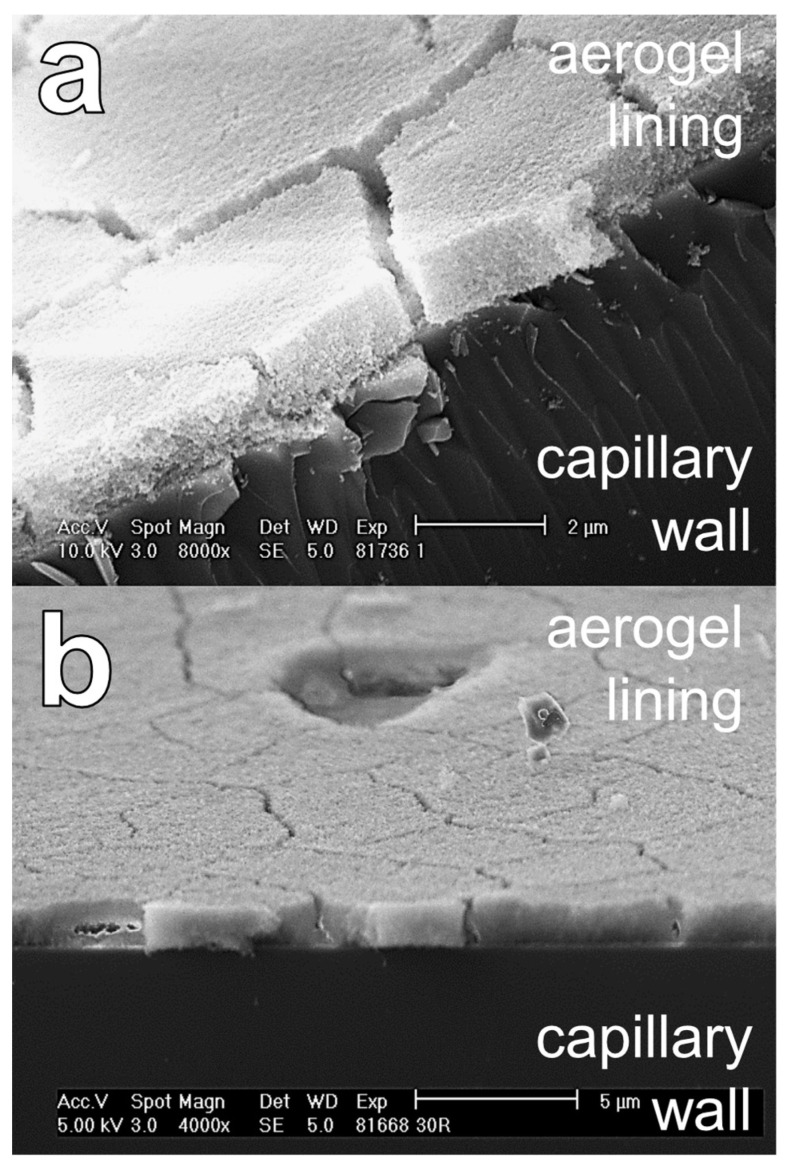
SEM images of aerogel linings featuring thicknesses of d > 1.0 µm, showing cracks (**a**,**b**) and holes (**b**).

The examination of ~200 SEM images revealed that aerogel linings of different thicknesses differed neither structurally nor topographically. However, the examination also revealed that the number of cracks per area increases with the thickness of the lining. Thus, the decrease in the Raman signal gain G for a lining thickness exceeding 1.0 µm may be due to the fact that cracks form in the thicker linings (see Figure 9a,b), which even can cause parts of the lining to detach and, thus, create holes (see Figure 9b). The fact that cracks in the aerogel lining can cause parts of the lining to detach also indicates that the adhesion of the aerogel lining to the capillary needs to be strengthened.

We cannot exclude that eventually the breakage of the capillaries (required for making the SEM images) resulted in the formation of cracks or holes in the lining. In future, we will, therefore, prepare the samples in such a way that the lining cannot be damaged (e.g., by cutting the capillaries with a laser). But we want to underline that we did not find cracks or holes in the linings of the broken capillaries that featured linings thinner than 1.0 µm. It is also documented in the literature that thin films of colloidal silica dispersions form cracks during drying if the thickness of the film exceeds a critical value [59]. However, we assume that a completely crack-free aerogel lining does not have to feature a maximum thickness but has to feature a minimum thickness ranging in the magnitude of the wavelength of the light to be guided (≥ 532 nm) in order to enable optimum Raman signal gain.

**Figure 10:**
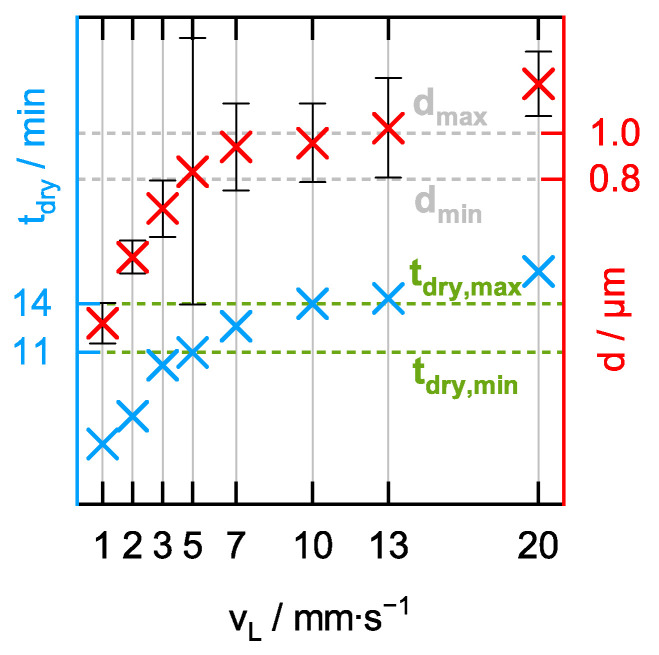
Drying time t_dry_ and aerogel lining thickness d as functions of the lining velocity v_L_.

Figure 10 compares the measured drying times t_dry_ of the same eight capillaries with their respective lining thicknesses d, plotted against the lining velocity v_L_. It can be seen that the thickness d of the aerogel lining increases with increasing drying time t_dry_. The thicker the aerogel lining is, the longer the drying time t_dry_. The range of optimum lining thickness d_min_ < d_opt_ < d_max_ resulting in optimum Raman signal gain G can, therefore, also be assigned a range of optimum drying time t_dry,min_ < t_dry,opt_ < t_dry,max_. This means that it is already possible to estimate during the drying process whether the alcogel-lined capillary currently being dried will have an optimum Raman signal gain G or not. This holds great potential for a manufacturer as it increases the efficiency of the manufacturing of aerogel-lined capillaries, as no further time-consuming quality control using Raman spectroscopy and SEM is required after the time-consuming drying step.

In a further experiment, we marked 40 equally long increments on the outer surface of a 20 cm long capillary lined with alcogel and measured the time required for the drying front to pass through each individual increment. We found that the time required for the drying front to pass the 0.5 cm long increment is similar for all 40 increments. Therefore, we assume that the thickness of the aerogel lining is approximately constant along a 20 cm aerogel-lined capillary. From this experiment, we furthermore conclude that during the process of air drying, the alcogel lining in the upright capillary does not flow towards the bottom of the capillary due to gravity.

**Figure 11:**
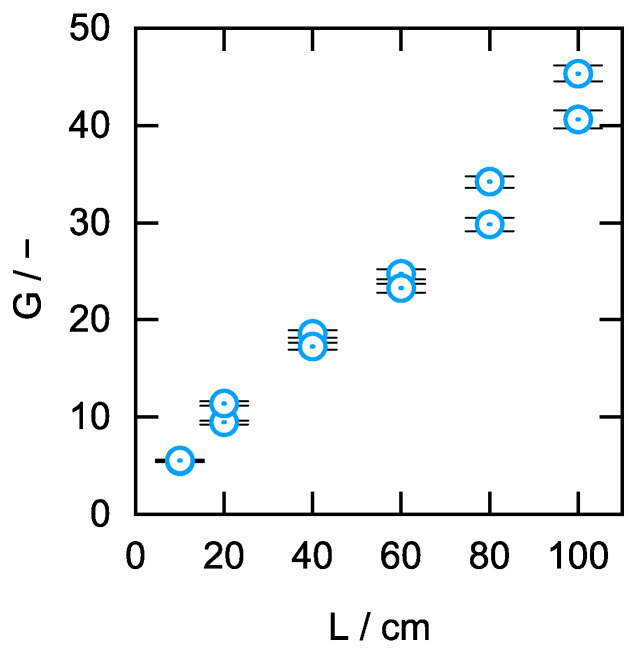
Raman signal gain G as a function of the length of the aerogel-lined capillary.

After the experimental determination of the range in which the lining velocity v_L_, the lining thickness d, and the drying time t_dry_ should be set to achieve an optimum Raman signal gain G, the final aim was to check whether or not the manufacturing process can also be used for the manufacturing of aerogel-lined capillaries of greater length, which offer an even higher Raman signal gain G. For this purpose, six capillary duplicates of different lengths ranging from L = 10 to 100 cm were manufactured in accordance with Section 2.1, Section 2.2 and Section 2.3, with the lining velocity v_L_ set to 7 mm/s. The Raman signal gain G of these twelve aerogel-lined capillaries was determined as described in Section 2.4. The determined values of the Raman signal gain G plotted against the respective lengths of the aerogel-lined capillaries are shown in Figure 11, in which the error bars represent the maximum error of G resulting from the propagation of the maximum errors from A_ALC_ and from A_CUV_. It becomes clear that the manufacturing process presented is suitable for manufacturing aerogel-lined capillaries whose Raman signal gain G can be scaled via the choice of capillary length within the tested frame.

In comparison to the aerogel-lined capillaries we manufactured earlier via spin-coating [51], the aerogel-lined capillaries we present here feature identical resistance against liquid, acidic, aqueous mixtures of organic substances.

## 4. Conclusions

We have presented an advanced process for manufacturing aerogel-lined capillaries suitable as LCWs that gain the Raman signal of pure liquid water (compared to a cuvette reference measurement). As shown in [51], these capillaries also gain the Raman signal of aqueous solutions. The obtained LCWs are, therefore, suitable for process analytics with rather low excitation light powers or short measurement times.

In order to avoid clogging of the LCW, it should not be used with liquids containing particles with diameters in the order of or exceeding the inner diameter of the LCW.

The presented manufacturing process makes it possible to predict—during the production of the aerogel lining—whether the resulting aerogel-lined capillary will feature optimum Raman signal gain or not. If not, the process can be optimized by adjusting just one parameter (the lining velocity), enabling a manufacturer of aerogel-lined capillaries to save time and money, especially when producing large batches of aerogel-lined capillaries.

Our study also shows that the Raman signal gain of an aerogel-lined capillary scales proportionally with the length of the capillary. This finding allows us to estimate the capillary length in order to achieve the Raman signal gain required for the particular measurement purpose.

Future work will aim at preventing the formation of cracks during the production of aerogel linings (e.g., using polymer additives [59]) and at strengthening the adhesion of the aerogel lining to the capillary wall, but also at the functionalization of the lining for capturing specific molecules.

## Data Availability

Data underlying the results presented in this paper are not publicly available at this time but may be obtained from the authors upon reasonable request.

## Supplementary Materials

The following supporting information can be downloaded at: https://www.mdpi.com/article/10.3390/s24185979/s1, Movie S1. Showing the opaque drying front moving in the direction of the air flow through the capillary during the conversion of the alcogel lining into the aerogel lining.
